# Editorial: The Evolving Landscape, Clinical Implications, and Future Perspective of Biomarkers in Gastrointestinal Cancers

**DOI:** 10.3389/fonc.2022.895231

**Published:** 2022-05-03

**Authors:** Hsiang-Lin Tsai, Yen-Cheng Chen, Kenneth KW To, Jaw-Yuan Wang

**Affiliations:** ^1^ Division of Colorectal Surgery, Department of Surgery, Kaohsiung Medical University Hospital, Kaohsiung Medical University, Kaohsiung, Taiwan; ^2^ Department of Surgery, Faculty of Medicine, College of Medicine, Kaohsiung Medical University, Kaohsiung, Taiwan; ^3^ Graduate Institute of Clinical Medicine, College of Medicine, Kaohsiung Medical University, Kaohsiung, Taiwan; ^4^ School of Pharmacy, Faculty of Medicine, The Chinese University of Hong Kong, Hong Kong, Hong Kong SAR, China; ^5^ Graduate Institute of Medicine, College of Medicine, Kaohsiung Medical University, Kaohsiung, Taiwan; ^6^ Center for Cancer Research, Kaohsiung Medical University, Kaohsiung, Taiwan; ^7^ Center for Liquid Biopsy and Cohort Research, Kaohsiung Medical University, Kaohsiung, Taiwan; ^8^ Pingtung Hospital, Ministry of Health and Welfare, Pingtung, Taiwan

**Keywords:** gastrointestinal cancers, biomarkers, evolving landscape, early detection, postoperative surveillance

Gastrointestinal (GI) cancer is a major public health problem worldwide. Early GI cancer detection, pretherapeutic responsiveness prediction, and postoperative micrometastasis monitoring are the hallmarks for successful GI cancer treatment. The approval of novel prognostic models and therapies for metastatic GI cancer (mGIC) has led to important improvements in patient outcomes.

Histocompatibility leukocyte antigen complex P5 (HCP5) is a momentous long non-coding RNA (lncRNA) and is involved in many autoimmune diseases and malignant tumors. Qin et al. demonstrated that the increase of serum HCP5 could significantly distinguish between patients with primary gastric cancer (GC) and healthy subjects, and the combined diagnosis of HCP5, CEA, and CA199 had high diagnostic efficiency.

Identification of a simplified prediction model for lymph node metastasis (LNM) for patients with early colorectal cancer (CRC) is urgently needed to determine treatment and follow-up strategies. CRC patients with tumor larger than 3 cm, who were identified as high-risk through the model, may require careful attention. Early CRC could be detected through the development of a novel prognostic model for predicting lymph node metastasis (Ahn et al.). Likewise, Log odds of positive lymph node scheme (LODDS) is an innovative N staging system and has been recently introduced as a new prognostic index in CRCs, which could powerfully stratify patients into different risk groups even when dissected lymph nodes were insufficient. Zhu et al. presented that a novel prognostic model incorporating common TBs (CA 199, CA125, and CEA) and LODDS displayed better predictive performance than both single factor and the TNM classification.

Systematic inflammatory factors, such as lymphocytes, monocytes, neutrophils, and blood biochemical indicators related to nutritional status, such as C-reactive protein (CRP) levels and albumin (ALB) levels, are valuable prognostic indicators for cancers including CRC. The prognostic value of new index (LANR) composed of pre-operative lymphocytes, albumin, and neutrophils has been demonstrated to be an important prognostic indicator for patients with resectable CRC (Liang et al.). Immunotherapy and induction of ferroptosis are both considered to be of great significance in clinical management of CRC. A ferroptosis-related genes model consisted of five genes: AKR1C1, ALOX12, CARS1, FDFT1, and ATP5MC3. This was built as a prognosis model for ferroptosis and might provide clues for further therapy in CRC (Nie et al.).

Various studies have suggested that the pathogenesis of CRC is influenced not only by genetic factors but also by altered gut microbial composition. Gut microbes can produce extracellular vesicles (EVs), also called nanovesicles, and are upregulated during cell activation and growth during cancer development. Profiling of microbe-derived EVs may offer a novel biomarker for detecting and predicting CRC prognosis (Park et al.).

Liquid biopsies allowing for individualized risk stratification of cancer patients have become of high significance in individualized cancer diagnostics and treatment. Ottaiano et al. contribute to a biological basis approach of *KRAS* testing with a more dynamic attitude (liquid biopsy) giving both new prognostic and therapeutic chances. Furthermore, they demonstrated that an association between *KRAS* regressive trajectory and the oligo-metastatic status was found and regressive and progressive mutational trajectories emerged as independent prognostic factors for survival. Hendricks et al. showed a novel immunofluorescence-based and a molecular detection approach for enumeration and detection of circulating tumor cells (CTCs). They enlighten the kinetics of CTC in CRC patients and support that the significance of CTC as a prognostic biomarker in a more in-depth longitudinal analysis of CTC over the course of the disease. Chen et al. reported that emerging technologies (liquid biopsy and exosomes media) may help early diagnosis of peritoneal metastasis by screening exosome miRNAs and exosomes-based treatment by transferring anti-tumor drugs and restricting exosomes homing in peritoneal metastasis.

Immune checkpoint blockade (ICB) shows remarkable clinical effects in patients with metastatic microsatellite-unstable (MSI) cancer. Busch et al. demonstrates that beta-2-microglobulin mutations are linked to a distant metastatic pattern and a favorable outcome in MSI stage IV GI cancers.

This editorial describes the novel biomarkers relevant to GI cancers. There is currently great focus on the discovery and validation of further biomarkers, with many new potential prognostic and predictive markers being identified alongside developments in molecular profiling technologies. Furthermore, the future perspective of emerging biomarkers’ development for the unmet medical need for GI cancer patients is mandatory.

## Author Contributions

H-LT and Y-CC contributed to the draft and writing; J-YW and KT reviewed the final version of this manuscript. All authors contributed to the article and approved the submitted version.

## Conflict of Interest

The authors declare that the research was conducted in the absence of any commercial or financial relationships that could be construed as a potential conflict of interest.

## Publisher’s Note

All claims expressed in this article are solely those of the authors and do not necessarily represent those of their affiliated organizations, or those of the publisher, the editors and the reviewers. Any product that may be evaluated in this article, or claim that may be made by its manufacturer, is not guaranteed or endorsed by the publisher.

